# Microbiome of the wasp *Vespula pensylvanica* in native and invasive populations, and associations with Moku virus

**DOI:** 10.1371/journal.pone.0255463

**Published:** 2021-07-29

**Authors:** Jason A. Rothman, Kevin J. Loope, Quinn S. McFrederick, Erin E. Wilson Rankin

**Affiliations:** 1 Department of Molecular Biology and Biochemistry, University of California: Irvine, Irvine, CA, United States of America; 2 Department of Biology, Georgia Southern University, Statesboro, GA, United States of America; 3 Department of Entomology, University of California: Riverside, Riverside, CA, United States of America; USDA Agricultural Research Service, UNITED STATES

## Abstract

Invasive species present a worldwide concern as competition and pathogen reservoirs for native species. Specifically, the invasive social wasp, *Vespula pensylvanica*, is native to western North America and has become naturalized in Hawaii, where it exerts pressures on native arthropod communities as a competitor and predator. As invasive species may alter the microbial and disease ecology of their introduced ranges, there is a need to understand the microbiomes and virology of social wasps. We used 16S rRNA gene sequencing to characterize the microbiome of *V*. *pensylvanica* samples pooled by colony across two geographically distinct ranges and found that wasps generally associate with taxa within the bacterial genera *Fructobacillus*, *Fructilactobacillus*, *Lactococcus*, *Leuconostoc*, and *Zymobacter*, and likely associate with environmentally-acquired bacteria. Furthermore, *V*. *pensylvanica* harbors—and in some cases were dominated by—many endosymbionts including *Wolbachia*, *Sodalis*, *Arsenophonus*, and *Rickettsia*, and were found to contain bee-associated taxa, likely due to scavenging on or predation upon honey bees. Next, we used reverse-transcriptase quantitative PCR to assay colony-level infection intensity for Moku virus (family: Iflaviridae), a recently-described disease that is known to infect multiple Hymenopteran species. While Moku virus was prevalent and in high titer, it did not associate with microbial diversity, indicating that the microbiome may not directly interact with Moku virus in *V*. *pensylvanica* in meaningful ways. Collectively, our results suggest that the invasive social wasp *V*. *pensylvanica* associates with a simple microbiome, may be infected with putative endosymbionts, likely acquires bacterial taxa from the environment and diet, and is often infected with Moku virus. Our results suggest that *V*. *pensylvanica*, like other invasive social insects, has the potential to act as a reservoir for bacteria pathogenic to other pollinators, though this requires experimental demonstration.

## Introduction

Social insects present some of the most widespread and damaging examples of invasive species worldwide [1]. Invasive ants, bees, and wasps exert outsized effects on the ecosystems they are introduced to—either by predation of, or competition with, native fauna [2–5]. Several species of yellowjacket wasps in the genus *Vespula* are particularly problematic in this respect [5]. During the 20^th^ century, members of the genus *Vespula* have become invasive in South America, New Zealand, Australia, South Africa, and a variety of islands where native arthropods have not coevolved with predatory vespines [6]. Given that these species are generalist predators, opportunistic scavengers, and often reach extreme densities in new environments, the effects on native arthropod communities have been profound. One example occurs in the Hawaiian Islands, where *Vespula pensylvanica* became widespread in the 1970s, introduced from its native range of western North America [7, 8]. Throughout most of the native range on the mainland, *V*. *pensylvanica* typically exhibits the annual life cycle common to most temperate social wasps and bees: colonies are founded by solitary queens in the spring, grow through the summer, and produce many dispersing males and daughter queens in the fall before dying [9]. Only hibernating new queens survive the winter. However, occasionally in the native range [10] and more commonly in subtropical Hawaii [7, 11], colonies survive the winter and attain tremendous sizes through a second or third season of growth [7]. These perennial colonies have an outsized effect on arthropod communities, collecting much more prey than typical annual colonies, and also exerting predation pressure throughout the year [11]. A prerequisite for extended colony lifespan is likely the adoption of multiple secondary queens, which is common in Hawaii [7, 12]. Foreign queen adoption may be facilitated by weak nestmate recognition in *V*. *pensylvanica* and porous colony boundaries, apparent in both the native and introduced range [13]. Understanding the ecological, genetic and social factors that have facilitated the successful Hawaiian invasion and the rise of the large and long-lived colony phenotype in Hawaii will be essential in mitigating the effects of the invasion and avoiding similar invasions elsewhere.

Invasive species potentially alter microbes present in their introduced environment [14], or the microbiomes of native species [15], and may harbor symbionts that allow these invasive species to exploit new resources [16]. Hymenoptera are often exposed to microbes through environmental contact [17–19], vertical transmission [20], or social transfer between nestmates [21, 22]. Within hymenopterans, there exists an interesting continuum of sociality [23] that allows for studies into the effects of lifestyle and host-microbe interactions. For example, social bees have a defined and consistent gut microbiome [21, 24], and some social ants (e.g., *Cephalotes* spp.) and wasps (*Vespa* and *Vespula* spp.) seem to have simple core microbiomes, although it is unknown how ubiquitous the taxa are between colonies [25–28]. Conversely, solitary Hymenoptera, generally associate with environmentally-acquired bacteria [17–19, 29–31], although there is evidence of megachilid, halictid, and apid solitary bees harboring some microbial taxa more than would likely be expected from solely environmental transmission [32, 33]. Furthermore, hymenopterans often associate with a variety of endosymbiotic bacteria that alter fitness, such as *Rickettsia*, *Arsenophonus*, and *Wolbachia* [20, 34–36], along with symbioses whose effects are unknown, such as that of *Sodalis* [37, 38]. Given that previous studies have found sequences corresponding to consumed prey bee-associated bacterial symbionts in other vespids [25], and that *V*. *pensylvanica* is native to the Western United States and is invasive to Hawaii [11], *V*. *pensylvanica* represents an excellent model species to understand the microbial ecology of isolated populations of invasive social wasps and possible mechanisms of microbial transmission.

Viral infections are widespread concerns for social Hymenoptera, and have been studied extensively in honey bees and *Vespula* spp. For example, honey bees harbor several debilitating viral diseases such as Deformed Wing Virus (DWV), Slow Bee Paralysis Virus (SBPV), Sacbrood Virus (SBV), Black Queen Cell Virus (BQCV), Kashmir Bee Virus (KBV), and others known to contribute to colony loss and impaired fitness (reviewed in [39]). While these are often thought of as “honey bee viruses,” infections in social wasps have been identified indicating the potential for cross-infection between insect species [28, 40–44]. Invasive species such as *V*. *pensylvanica* may spread diseases to new ranges and novel hosts, or conversely, be infected by endemic diseases that are commonly found in newly sympatric hosts [28, 40, 44]. In order to study the effects of emerging infectious diseases on insect populations, we surveyed *V*. *pensylvanica* colonies for Moku virus—a recently-described single-stranded positive-sense RNA virus (family: Iflaviridae) originally described in wasp colonies in Hawaii [45]. Aside from Hawaii, Moku virus has been found in *Vespula* spp. from several geographical locations, and has been found in honey bees and their associated *Varroa* mites [28, 45, 46]. While Moku virus is apparently widespread and may infect multiple insect species, the transmission potential, virulence, and effects on insect populations of Moku infections are currently unknown, although it is hypothesized that *Vespula* spp. are the natural reservoirs of this disease [45]. Previous work in our study population of *V*. *pensylvanica* suggests that Moku virus loads are strongly bimodal at the colony level, that colonies with high loads have reduced longevity in some years, and colonies that are at higher densities have higher Moku loads [47].

As the microbial ecology and effects of Moku virus on *V*. *pensylvanica* are largely unknown, we conducted an exploratory study using 16S rRNA gene sequencing and RT-qPCR on these invasive social wasps at the colony level. We investigated three lines of inquiry to better understand the microbial communities of our study organism: First, does *V*. *pensylvanica* associate with a defined microbiome, is there any evidence of environmental transmission of microbes, and are their microbiomes similar in the native and invasive range? Second, does this wasp species associate with endosymbiotic bacteria? Third, are there associations between Moku virus and the wasp microbiome?

## Materials and methods

### Field sites and collections

Fieldwork was conducted under a permit from the National Park Service (HAVO-2016-SCI-0050). We collected adult worker wasps from the entrances of colonies found along Hilina Pali Road and at Kipuka Kahali’i in Hawaii Volcanoes National Park (HAVO) on the Big Island of Hawaii and the campus of the University of California, Riverside (UCR) in Riverside, CA. Colonies were discovered by following foragers back to the nest site, or by noticing characteristic foraging traffic at the colony entrance. We collected between 20 and 40 adult worker wasps from each of 40 colonies at HAVO between August 25–27, 2017, as well as from 13 colonies at UCR between October 6–26, 2017 (see S1 Table for nest site coordinates and sampling dates). We collected *V*. *pensylvanica* workers from nest entrances using a net or a portable vacuum. While collecting, we attempted not to disturb the colony and thus primarily collected foragers, though in some cases we likely collected guard wasps as well. We flash-froze the collected adults alive in a liquid nitrogen dry shipper, and then transferred to a -80°C freezer upon return to the lab until processing. Thirty-seven colonies sampled in Hawaii are included in a companion study on colony longevity in *V*. *pensylvanica* [47]. Three of the Hawaii colonies were likely perennial colonies that had survived from the previous year, given their size, and none of the monitored colonies in Hawaii survived the next winter.

### DNA extractions and library preparation

We extracted DNA by first pooling 20 wasps per colony (N = 53 colonies). We homogenized the samples by adding still-frozen insects to 15 mL tubes with grinding balls using a Geno/Grinder (SPEX SamplePrep, Metuchen, NJ), with the block pre-chilled with liquid nitrogen. We then quickly transferred the homogenate to new sample tubes and re-froze at -80°C until DNA or RNA extraction. Ultimately, we extracted DNA from a ~20 mg aliquot of thawed homogenate using a Qiagen DNeasy kit (Qiagen, Valencia, CA) by following the manufacturer’s protocol plus an overnight incubation at 55°C.

We prepared paired-end 16S rRNA gene libraries for samples (N = 53) using a protocol based on Engel et al. [48], Rothman et al. 2018 [49] and 2020 [50], and McFrederick and Rehan 2016 [51]. Briefly, we generated libraries with a universal 16S primer sequence corresponding to the region 799–1115 (799F-mod3: CMGGATTAGATACCCKGG and 1115R: AGGGTTGCGCTCGTTG) chosen to avoid plant plastid contamination [52, 53], a unique barcode sequence, and Illumina adapter sequence through two rounds of PCR. We normalized the resulting libraries with a SequalPrep Normalization kit (ThermoFisher, Waltham, MA), then pooled the libraries and performed a final cleanup with a PureLink PCR Purification kit (Invitrogen, Carlsbad, CA). Finally, we sequenced the libraries with a V3 Reagent Kit with 2 X 300 cycles on an Illumina MiSeq Sequencer in the UC Riverside Institute for Integrated Genome Biology. We also ran blank samples to control for reagent contamination that we prepared and sequenced in the same way as regular samples. Raw sequence data are available on the NCBI Sequence Read Archive under accession number PRJNA707052.

### Quantification of Moku viral titer

We used data reported in another study on Moku Virus and colony longevity [47] and repeat the methods here for clarity. Briefly, we quantified Moku virus titer using reverse-transcription quantitative PCR (RT-qPCR) of viral RNA. For each colony, we extracted RNA from a ~20 mg aliquot of re-frozen wasp homogenate from 20 pooled workers (the homogenization procedure is described above) using 1 ml of TRIsure (Bioline Inc., Taunton, MA) following the manufacturer’s protocols. We then quantified RNA for each sample using a Qubit spectrophotometer (ThermoFisher Scientific, Waltham, MA), and normalized RNA concentration to 2ng/ul. We then amplified Moku virus RNA using the primers MVF and MVR [46], and amplified *Vespula eIF3* [54] as a reference gene. We used a BioRad CFX Real Time PCR machine (BioRad, Hercules, CA), and Luna OneStep RT-qPCR kits (New England Biolabs, Ipswich, MA) according to manufacturer instructions, with 10ul reaction volumes, 10ng of RNA per reaction, and an annealing temperature of 60°C. Melt curves were checked to verify a single PCR product. We quantified reaction efficiencies alongside samples, and always obtained efficiencies between 95 and 105% for the 10-fold dilution series spanning the range of observed sample Cq values. We ran all samples in duplicate, and averaged Cq values. We calculated relative Moku Virus titer as–(Cq_moku_-Cq_*eIF3*_) for each sample, resulting in an index with higher values corresponding to greater viral titer on a log scale. Because colony virus titer was strongly bimodal in a larger dataset including two years of data [47], we used a titer of 7 as a threshold and classified colonies as either high-titer or low-titer.

### Bioinformatics and statistics

We processed the 16S rRNA gene libraries with the QIIME2 pipeline (v2019.7) [55] by first trimming adapters, sequencing primers, and low-quality ends from the reads, then used DADA2 [56] to quality filter the reads, remove singletons, and bin sequences into Amplicon Sequence Variants (ASVs; 100% identical sequence reads) using the default parameters, followed by reagent contamination removal with the R package “decontam” [57]. We used the QIIME2 q2-feature classifier [58] to assign taxonomy to the ASVs with the SILVA database trained to the 799–1115 region of the 16S rRNA gene [59] (as well as local BLAST searches against the NCBI 16S Microbial database), and generated sequence alignments with MAFFT [60], then tabulated ASV counts in a table. We used this table to calculate diversity metrics, and tested the statistical significance of alpha diversity (Shannon Diversity Index) with Kruskal-Wallis tests in QIIME2, and beta diversity (Bray-Curtis dissimilarities) through Adonis PERMANOVA (999 permutations) with the R package “vegan” [61] in R v. 3.5.1 [62]. We visualized the Shannon diversities through boxplots, proportional abundance of bacterial taxa as a stacked bar plot, and beta diversity through Non-metric Multidimensional Scaling (NMDS) with the R package “ggplot2” [63]. We indicated shared and unique ASVs through Venn diagrams with the BEG Venn Diagram tool (http://bioinformatics.psb.ugent.be), and plotted heatmaps with “pheatmap” [64]. We used ANCOM [65] to test for differentially abundant taxa between locations, and used mantel tests in “vegan” to test for correlations between bacterial diversity and Moku virus titer.

## Results

**Fig 1:**
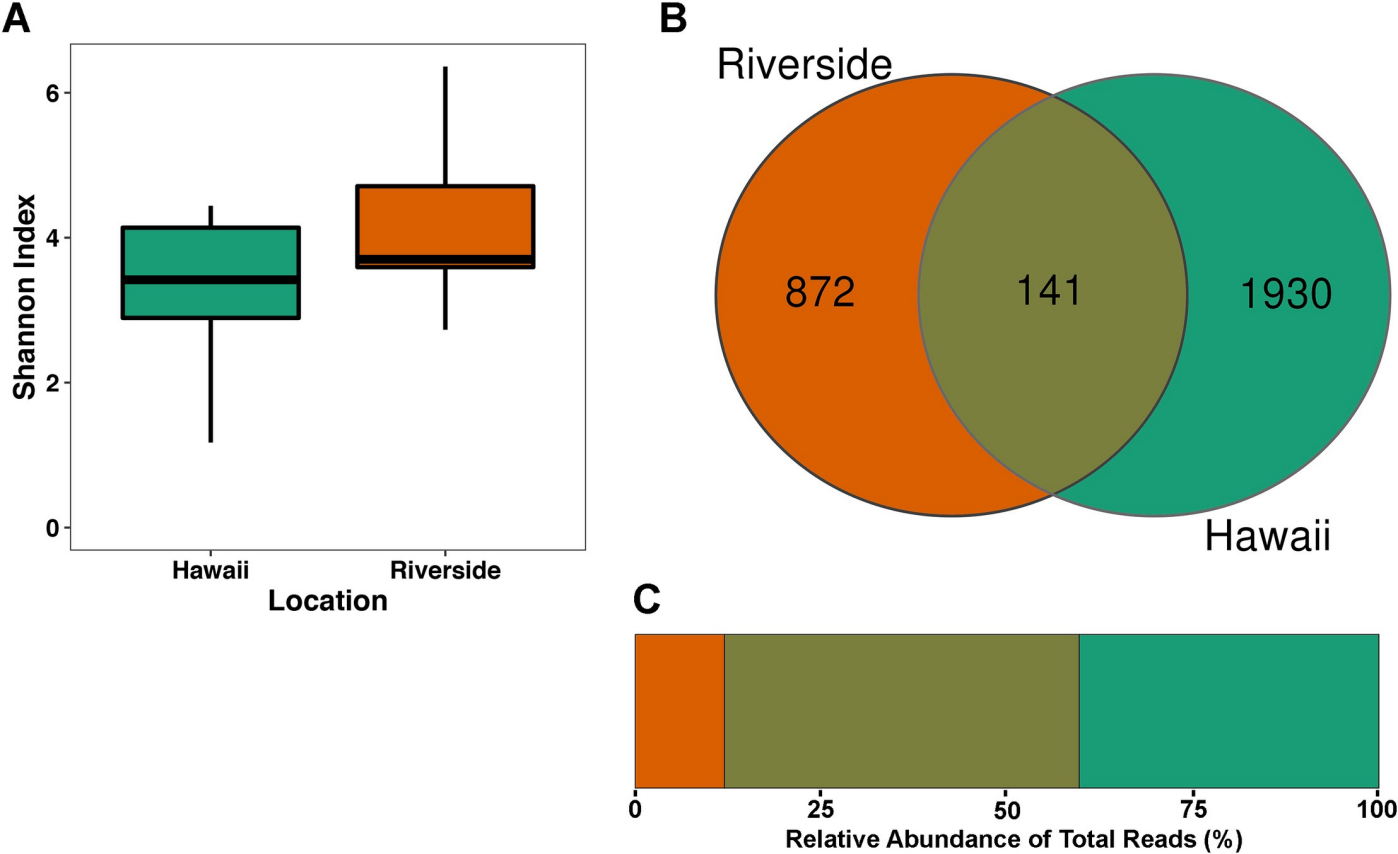
A) Boxplot of the Shannon diversities of wasp microbial communities from Riverside or Hawaii, which were not shown to be significantly different (H = 2.62, P = 0.11). B) Venn diagram showing the number of bacterial ASVs shared or unique between Hawaii and Riverside samples. C) Stacked barplot indicating the proportion of total reads shared or unique between Hawaii and Riverside samples.

We obtained 1,033,410 quality-filtered (average quality score Q38) 16S rRNA gene sequences with an average of 19,498 reads per sample from two separate populations (Total N = 53; “Riverside” N = 13; “Hawaii” N = 40) that clustered into 2,943 unique ASVs (S1 File). Through rarefaction analyses, we determined that we had acceptable ASV coverage at a read depth of 2,232 reads (S1 Fig), which left us with N = 45 samples (Riverside, CA [N = 12], Volcanoes, HI [N = 33]). We compared the Shannon diversity of our samples, but did not find a significant difference in alpha diversity between the two populations (H = 2.62, P = 0.11, Fig 1). Most of the ASVs were unique to each population, with 872 (29.6% of ASVs, 12.0% of reads) only found in Riverside samples, 1,930 (65.6% of ASVs, 40.3% of reads) only found in Hawaiian samples, and 141 (4.8% of ASVs, 47.7% of reads) found in both populations (Fig 1).

**Fig 2:**
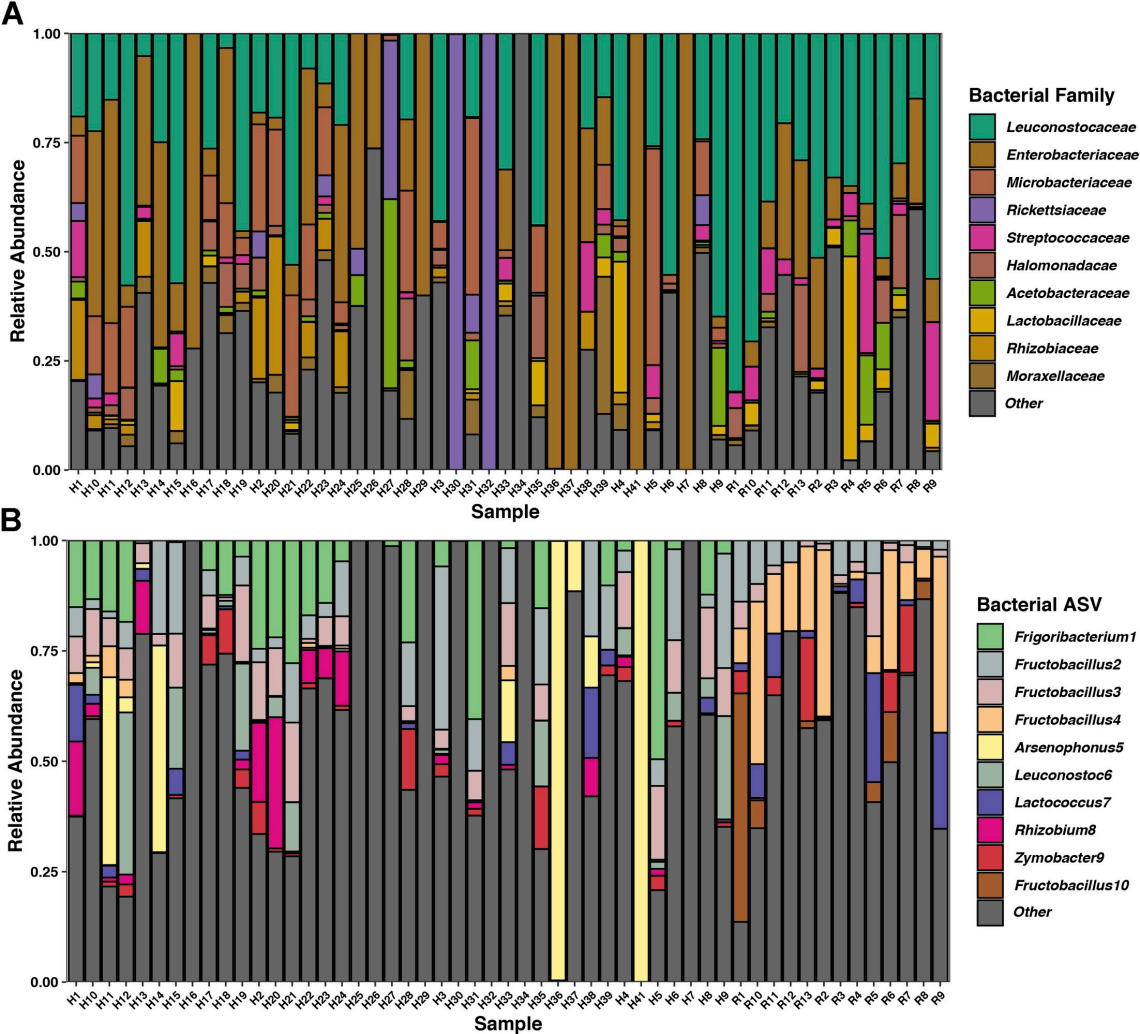
Stacked bar plot of the top ten overall most proportionally abundant bacterial A) families and B) Amplicon Sequence Variants (ASVs) in our samples. The letters “H” or “R” denotes Hawaiian or Riverside sampling locations.

**Fig 3:**
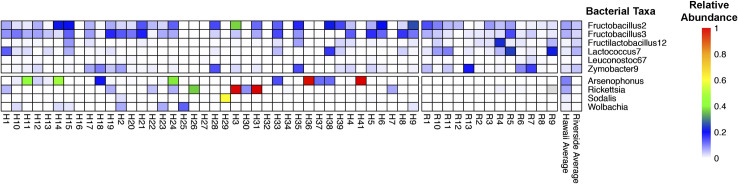
Heatmap of the proportional abundances of Amplicon Sequence Variants (ASVs) identified in at least 50% of our samples and in both locations, and genera that have been identified in other studies as potential insect symbionts. Rows are individual ASVs or genera, columns are individual samples, and heat color represents proportional abundance of these taxa. Final panel is the mean proportional abundance of each taxon in either Hawaiian or Riverside samples.

After removing likely reagent contamination identified in our control blanks (generally *Pseudomonas* spp, *Shewanella* spp., *Halomonas* spp., *Micrococcus* spp., *Cutibacterium acnes*, *Streptococcus oralis*, and Rhizobia), the top 10 most abundant bacterial families and their relative abundances in our samples were as follows: Leuconostocaceae (32.8%), Enterobacteriaceae (17.1%), Microbacteriaceae (9.9%), Streptococcaceae (3.6%), Lactobacillaceae (3.6%), Halomonadacae (3.1%), Rhizobiaceae (2.9%), Acetobacteraceae (2.6%), Rickettsiaceae (2.4%), and Moraxellaceae (1.9%), while all other families combined comprised an average of 20.2% of the proportional abundance of taxa (Fig 2). Likewise, while our samples contained many individual ASVs, they were dominated by relatively few. The top 10 most abundant ASVs were: a *Frigoribacterium faeni* ASV (9.7%), four *Fructobacillus* ASVs (*F*. *fructosus* [7.8%], *F*. *fructosus* [7.1%], and two unknown *Fructobacillus* spp. [6.3% and 2.5%]), an *Arsenophonus* sp. ASV (6.3%), a *Leuconostoc* sp. ASV (3.7%), an ASV of *Lactococcus lactis* (2.9%), a *Rhizobium* sp. ASV (2.7%), and a *Zymobacter palmae* ASV (2.5%), which cumulatively comprise 51.6% of the wasp microbial communities (Fig 2). Lastly, seven individual ASVs were both in greater than 50% of total samples *and* in both Riverside and Hawaiian populations, indicating at least some commonality between two geographically distinct populations of wasps: Two ASVs of *F*. *fructosus*, an ASV of *Zymobacter palmae*, an ASV of *Lactococcus lactis*, an ASV of *Fructilactobacillus vespulae*, and an unknown *Leuconostoc* ASV (Fig 3). We also found several bacterial genera associated with wasp symbiosis, and plot the relative abundances of *Arsenophonus*, *Rickettsia*, *Sodalis*, and *Wolbachia* in Fig 3.

**Fig 4:**
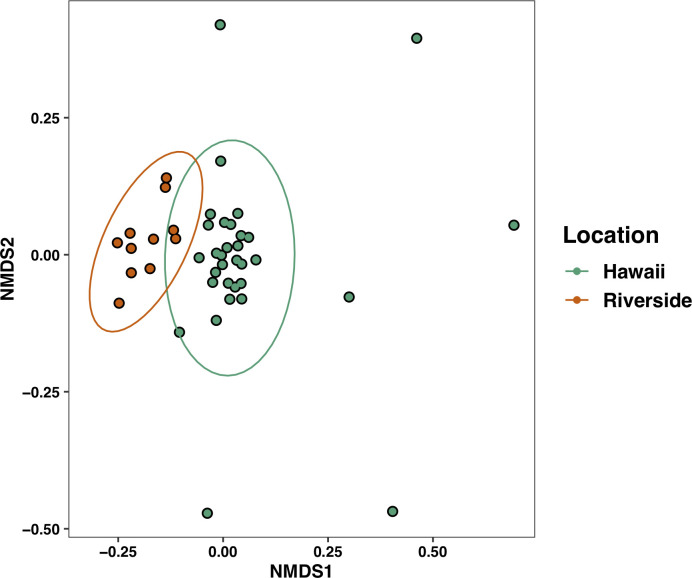
Non-metric multidimensional scaling (NMDS) plot of the Bray-Curtis dissimilarities of samples between Riverside and Hawaiian locations. Sampling location significantly affected the wasp microbiome (F = 5.9, R^2^ = 0.12, P < 0.001).

**Fig 5:**
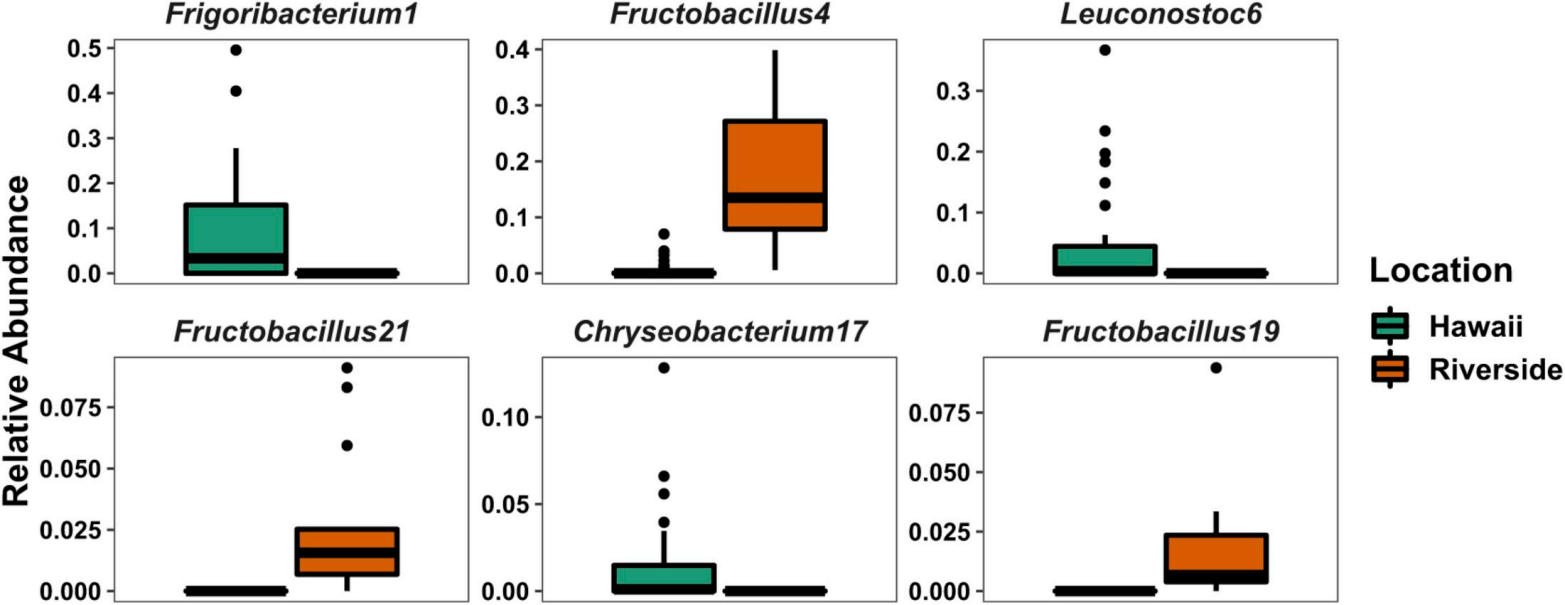
Boxplots showing the relative abundances of bacterial ASVs at greater than 0.5% overall relative abundance that were differentially abundant between Riverside and Hawaiian locations as tested with ANCOM.

We used Adonis PERMANOVA to investigate the effects of geographical location on the beta diversity of *V*. *pensylvanica*. We saw a significant difference in the diversity of bacteria in wasps collected from Riverside versus Hawaii (F = 5.9, R^2^ = 0.12, P < 0.001; Fig 4), then used ANCOM to test for differentially abundant bacterial ASVs at greater than 0.5% relative abundance between the two locations (Wald > 25; we note that several of the differentially abundant ASVs were found only in one site, Fig 5).

We compared the microbiomes of wasp colonies with low Moku virus titer (N = 7) or high Moku virus titer (N = 26) in our Hawaiian samples (we did not detect Moku virus in any Riverside samples). We found no significant effect of viral titer on either the alpha (H = 2.95, P = 0.09) or beta diversity (F = 0.99, R^2^ = 0.03, P = 0.45) in our samples, and did not find any significantly differentially abundant ASVs between high-titer and low-titer colonies. We also ran mantel tests using Spearman correlations to test relationships between wasps’ alpha (Shannon diversity index) and beta (Bray-Curtis dissimilarities) microbial diversity with Moku virus titer, but Moku virus titer was not significantly correlated with microbial alpha or beta diversity in our samples (alpha: ρ = -0.20, P = 0.32; beta: ρ = 0.07, P = 0.41).

## Discussion

Our results suggest that the invasive wasp *V*. *pensylvanica* associates with a simple microbiome consisting largely of lactic acid bacteria and *Zymobacter*, along with significant associations of endosymbiotic bacteria. Notably, by comparing wasp colonies from two geographically-distinct locations, we also show that social wasp-associated microbial communities may contain environmentally-associated microbes similar to other Hymenoptera [17, 18, 29, 50], and as mentioned above, wasp colonies appear to harbor some identical microbial taxa across their range. Similar to our findings, previous work has shown that some social *Vespa* species also possess simple microbiomes and associate with prey bees’ bacterial symbionts [25]. In our small-scale study, we found that the microbiomes of *V*. *pensylvanica* colonies are unaffected by Moku infection, and a larger sample size would be useful to confirm this result. Coupled with the fact that Moku virus is transmissible to honey bees [45, 46], multispecies infection interactions may occur between wasps and other insects. More research is needed to understand how ecological invasions affect insect populations and their associated microbes.

The microbial communities of wasp colonies in our study were somewhat different based on their geographical location, suggesting that environmental exposure affects which bacteria they associate with. For example, *Fructobacillus* spp. and *Leuconostoc* spp.–taxa known to associate with hymenopterans and flowers [17, 51, 66]—were significantly different between Hawaii and Riverside samples, and may be potential symbionts for insects or plants. Our results may also be affected by uneven sampling, which may have caused us to find more total ASVs in wasps from Hawaii simply due to a greater number of these samples. Interestingly, while environment apparently plays a significant role in bacterial inoculation, the wasps seem to associate with taxa conserved across geographic distances, consisting largely of members of the Lactobacillaceae family along with an ASV of *Zymobacter palmae*. While we are unable to conclude that this microbiome is maintained through generations, previous work has shown that other social wasps consistently harbored *Z*. *palmae*, and seemed to possess a small microbial community [25], although we recognize that we sequenced a different region of the 16S rRNA gene, potentially limiting direct comparisons with other amplicon studies. Likewise, as we analyzed pooled, whole wasps, we may be detecting bacteria present on the wasps and only reflect their surroundings, not a potential symbiosis. Similarly, we found taxa corresponding to honey bee gut microbiota suggesting that wasps were preying upon honey bees and were exposed to their bacteria, confirming previous results [25]. While this agrees with previous studies, the honey bee core bacteria were not seen ubiquitously throughout our samples, suggesting that the bacteria are not colonizing the wasps and are likely being detected only as DNA presence, not live cells. This result was not entirely surprising, as many members of the honey bee core gut bacteria are coevolved, specialized taxa that are unlikely to colonize other species [67]. As there is interest in the ability of wasps to vector diseases, assaying the specific anatomical locations and viability of wasp- and bee-associated bacteria present in *V*. *pensylvanica* would help uncover more interactions between invasive and native wasps.

Wasps in our study harbored a variety of putatively endosymbiotic bacteria—especially those collected in Hawaii. These endosymbionts included bacteria in the genera *Arsenophonus*, *Rickettsia*, *Sodalis*, and *Wolbachia*, which have been found in other wasp species [44, 68, 69], but to the best of our knowledge, we are the first to find *Rickettsia* and *Sodalis* spp. in *Vespula* species. Interestingly, several of our samples’ microbiomes were dominated by either *Arsenophonus* or *Rickettsia* implying that these bacteria are likely able to either outcompete other environmentally-acquired taxa or conserved microbes or reach such high densities that they dominate the sequencing results. While our results are promising, we are unable to rule out sampling/sequencing error as a source of the peculiarly high proportion of endosymbionts, and as our samples were pooled, we may simply be detecting a colony-wide infection rather than individual wasps’ microbes. We are also unable to track the transmission mode of the endosymbionts, especially as some are known to have complex epidemiology [70]. Additionally, in other samples, endosymbiont infections were either not detected or in lower proportional abundance as part of a more complex microbial community, especially when comparing the much lower levels in wasps collected in Riverside versus Hawaii. These two apparently conflicting results suggest that endosymbionts are not ubiquitous in *V*. *pensylvanica* colonies, and as the wasp colonies otherwise appeared normal, may not be virulent or beneficial to colonies. As our data are compositional, we are unable to assess the bacterial load of the endosymbionts at this time, as infection titer is likely important in understanding infection dynamics [71]. Suggestively, colony-level *Arsenophonus* titer (quantified with qPCR) was correlated with the proximity of wasp colonies to honey bee hives at the Hawaii field site [47], which could imply spillover from honey bees, although this remains to be tested. We suggest that manipulation studies be conducted with endosymbiotic bacteria to probe deeper into both individual- and colony-level effects on wasps in multiple generations.

Neither Moku virus presence nor titer affected the wasps’ microbiomes. Moku virus is a recently-discovered virus that is known to infect *V*. *pensylvanica* and honey bees, and has been previously detected in Hawaii, the United Kingdom, and Belgium [45, 46]—although the virulence, infectivity, and range of this virus is currently unknown. Moku virus titer predicted *Vespula* colony longevity in 2016 at our Hawaii field site, but not in 2017 when the samples in this study were collected, suggesting a variable role of this virus in wasp colony dynamics [47]. Moku virus presence and titer were not associated with microbiome diversity, indicating that there may not be cross-talk between the microbiome and this particular viral infection in social wasps. Likewise, as a non-phage virus, Moku likely does not directly infect bacteria [45]. Many other non-viral hymenopteran diseases are known to interact with the microbiome [72, 73], and in bees the gut microbiome is heavily involved in immune function and defense against parasites [73–76]. Even though Moku virus did not affect the microbiome, this virus can still infect honey bees, which indicates the potential for introduced wasps to transmit disease to nontarget hosts [44, 77].

In summary, our data suggest that the social wasp *V*. *pensylvanica* possesses a simple microbiome mainly composed of lactic acid bacteria and putatively environmentally-acquired taxa along with several species of endosymbionts. Furthermore, we show that these invasive wasps likely maintain some of these core bacteria across geographically distinct regions, indicating potential symbioses. We also found that wasp colony microbiomes are not affected by Moku virus presence, despite high titers, which supports the hypothesis of *V*. *pensylvanica* being a natural reservoir for this disease, along with a potential vector to other insects. We suggest that future studies examine the physiological effects of Moku virus on wasps and other Hymenoptera, and the potential for invasive wasps to change the microbial and viral ecology of their introduced ranges. We are also interested in assaying the contributions of wasps’ microbes to colony fitness and nutrition and subsequent work should be conducted to understand the microbial ecology of social wasps.

## Supporting information

S1 Fig Rarefaction curves of the 16S rRNA gene sequences for all samples. Curves saturate before a sequencing depth of 2,232 reads, which we used for all diversity analyses.
(DOCX)

S1 File Amplicon Sequence Variant (ASV) file containing counts per ASV per sample, SILVA taxonomy, and unique ASV identifier. (TXT)

S1 Table Latitude and longitude coordinates and sampling dates for each sample. “R” denotes “Riverside” and “H” denotes Hawaii sampling locations.
(DOCX)
