# Supplementary material for: Microbiome of the wasp Vespula pensylvanica in native and invasive populations, and associations with Moku virus
Source: PLoS One. 2021 Jul 29;16(7):e0255463. doi: 10.1371/journal.pone.0255463 (PMC8321129; doi:10.1371/journal.pone.0255463)
Supplement: S1 Fig — Curves saturate before a sequencing depth of 2,232 reads, which we used for all diversity analyses. (DOCX) [file pone.0255463.s001.docx]

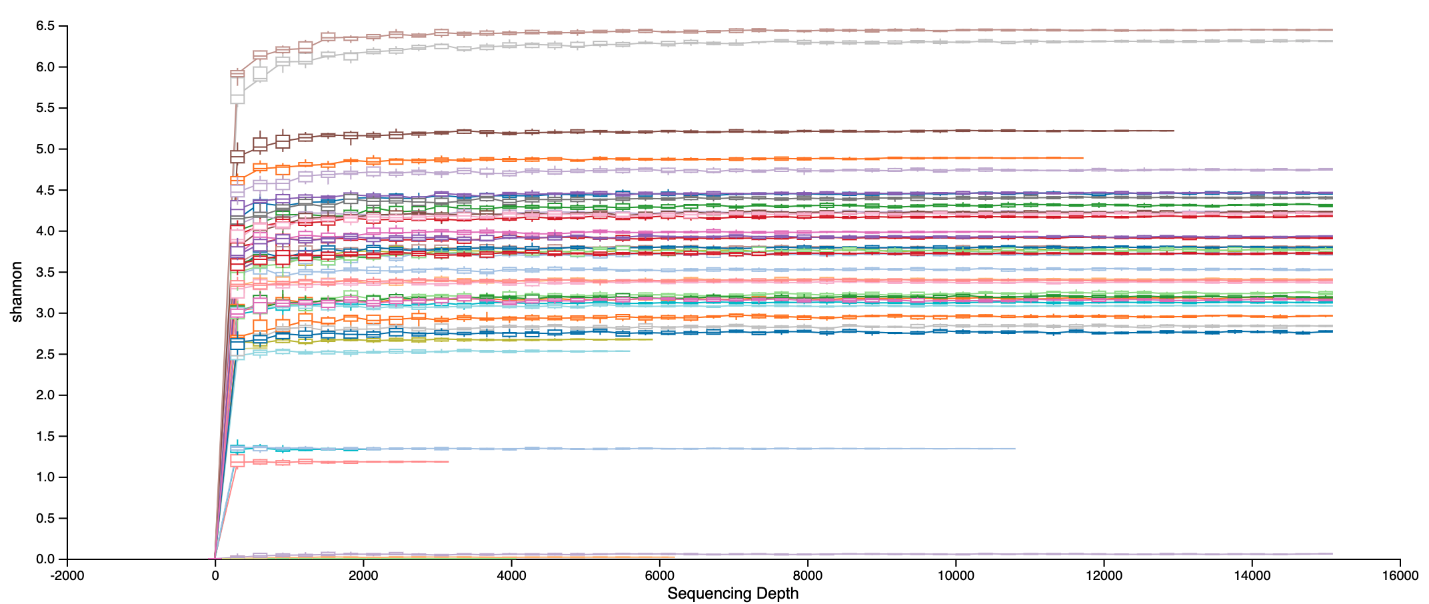


S1 Fig: Rarefaction curves of the 16S rRNA gene sequences for all samples. Curves saturate before a sequencing depth of 2,232 reads, which we used for all diversity analyses.
