# Supplementary material for: Microbiome of the wasp Vespula pensylvanica in native and invasive populations, and associations with Moku virus
Source: PLoS One. 2021 Jul 29;16(7):e0255463. doi: 10.1371/journal.pone.0255463 (PMC8321129; doi:10.1371/journal.pone.0255463)
Supplement: S1 Table — “R” denotes “Riverside” and “H” denotes Hawaii sampling locations. (DOCX) [file pone.0255463.s003.docx]

| Sample Name | Latitude/Longitude | Collection date |
| --- | --- | --- |
| H1 | 19.345 N 155.264 W | August 25-27, 2017 |
| H10 | 19.344 N 155.276 W | August 25-27, 2017 |
| H11 | 19.344 N 155.274 W | August 25-27, 2017 |
| H12 | 19.345 N 155.276 W | August 25-27, 2017 |
| H13 | 19.319 N 155.292 W | August 25-27, 2017 |
| H14 | 19.315 N 155.297 W | August 25-27, 2017 |
| H15 | 19.357 N 155.224 W | August 25-27, 2017 |
| H16 | 19.353 N 155.225 W | August 25-27, 2017 |
| H17 | 19.349 N 155.226 W | August 25-27, 2017 |
| H18 | 19.341 N 155.213 W | August 25-27, 2017 |
| H19 | 19.345 N 155.269 W | August 25-27, 2017 |
| H2 | 19.345 N 155.267 W | August 25-27, 2017 |
| H20 | 19.345 N 155.271 W | August 25-27, 2017 |
| H21 | 19.354 N 155.253 W | August 25-27, 2017 |
| H22 | 19.348 N 155.268 W | August 25-27, 2017 |
| H23 | 19.35 N 155.268 W | August 25-27, 2017 |
| H24 | 19.349 N 155.269 W | August 25-27, 2017 |
| H25 | 19.348 N 155.27 W | August 25-27, 2017 |
| H26 | 19.351 N 155.26 W | August 25-27, 2017 |
| H27 | 19.364 N 155.249 W | August 25-27, 2017 |
| H28 | 19.364 N 155.249 W | August 25-27, 2017 |
| H29 | 19.349 N 155.261 W | August 25-27, 2017 |
| H3 | 19.349 N 155.261 W | August 25-27, 2017 |
| H30 | 19.348 N 155.262 W | August 25-27, 2017 |
| H31 | 19.351 N 155.257 W | August 25-27, 2017 |
| H32 | 19.336 N 155.278 W | August 25-27, 2017 |
| H33 | 19.335 N 155.278 W | August 25-27, 2017 |
| H34 | 19.336 N 155.278 W | August 25-27, 2017 |
| H35 | 19.341 N 155.209 W | August 25-27, 2017 |
| H36 | 19.311 N 155.298 W | August 25-27, 2017 |
| H37 | 19.312 N 155.298 W | August 25-27, 2017 |
| H38 | 19.311 N 155.296 W | August 25-27, 2017 |
| H39 | 19.345 N 155.212 W | August 25-27, 2017 |
| H4 | 19.352 N 155.255 W | August 25-27, 2017 |
| H41 | 19.312 N 155.298 W | August 25-27, 2017 |
| H5 | 19.356 N 155.255 W | August 25-27, 2017 |
| H6 | 19.355 N 155.256 W | August 25-27, 2017 |
| H7 | 19.345 N 155.268 W | August 25-27, 2017 |
| H8 | 19.345 N 155.212 W | August 25-27, 2017 |
| H9 | 19.345 N 155.212 W | August 25-27, 2017 |
| R1 | 33.969 N 117.322 W | October 26, 2017 |
| R10 | 33.977 N 117.325 W | October 6, 2017 |
| R11 | 33.972 N 117.322 W | October 6, 2017 |
| R12 | 33.969 N 117.323 W | October 6, 2017 |
| R13 | 33.973 N 117.324 W | October 6, 2017 |
| R2 | 33.974 N 117.325 W | October 6, 2017 |
| R3 | 33.974 N 117.322 W | October 6, 2017 |
| R4 | 33.977 N 117.326 W | October 6, 2017 |
| R5 | 33.972 N 117.322 W | October 6, 2017 |
| R6 | 33.971 N 117.321 W | October 6, 2017 |
| R7 | 33.971 N 117.321 W | October 25, 2017 |
| R8 | 33.97 N 117.323 W | October 25, 2017 |
| R9 | 33.975 N 117.323 W | October 26, 2017 |

Table ST1: Latitude and longitude coordinates and collection date for each sample. “R” denotes “Riverside” and “H” denotes Hawaii sampling locations.
